# Evaluation on prognostic efficacy of lymph nodes ratio (LNR) and log odds of positive lymph nodes (LODDS) in complicated colon cancer: the first study in emergency surgery

**DOI:** 10.1186/s12957-018-1483-6

**Published:** 2018-09-13

**Authors:** Savino Occhionorelli, Dario Andreotti, Pierpaola Vallese, Lucia Morganti, Domenico Lacavalla, Elena Forini, Giovanni Pascale

**Affiliations:** 1 Department of Morphology, Surgery and Experimental Medicine, University of Ferrara, via A. Moro, 8, Cona, 44124 Ferrara, Italy; 2 Department of Oncological and Gastroenterological Surgical Sciences, University of Padova, Padua, Italy; 3 Unit of General Surgery, State Hospital of San Marino, Borgo Maggiore, Republic of San Marino; 4 Unit of Statistics, S. Anna University Hospital of Ferrara, Ferrara, Italy

**Keywords:** Colon cancer, Lymph nodes, LODDS, Survival analysis

## Abstract

**Background:**

Lymph node involvement is one of the most important prognostic factors in colon cancer. Twelve is considered the minimum number of lymph nodes necessary to retain reliable tumour staging, but several factors can potentially influence the lymph node harvesting. Emergent surgery for complicated colon cancer (perforation, occlusion, bleeding) could represent an obstacle to reach the benchmark of 12 nodes with an accurate lymphadenectomy. So, an efficient classification system of lymphatic involvement is crucial to define the prognosis, the indication to adjuvant therapy and the follow-up. This is the first study with the aim to evaluate the efficacy of lymph nodes ratio (LNR) and log odds of positive lymph nodes (LODDS) in the prognostic assessment of patients who undergo to urgent surgery for complicated colonic cancer.

**Methods:**

This is a retrospective study carried out on patients who underwent urgent colonic resection for complicated cancer (occlusion, perforation, bleeding, sepsis). We collected clinical, pathological and follow-up data of 320 patients. Two hundred two patients met the inclusion criteria and were distributed into three groups according to parameter N of TNM, LNR and LODDS. Survival analysis was performed by Kaplan-Meier curves, investigating both overall survival (OS) and disease-free survival (DFS).

**Results:**

The median number of harvested lymph nodes was 17. In 78.71% (*n* = 159) of cases, at least 12 lymph nodes were examined. Regarding OS, significant differences from survival curves emerged for ASA score, surgical indication, tumour grading, T parameter, tumour stage, N parameter, LNR and LODDS. In multivariate analysis, only LODDS was found to be an independent prognostic factor.

Concerning DFS, we found significant differences between survival curves of sex, surgical indication, T parameter, tumour stage, N parameter, LNR and LODDS, but none of these confirmed its prognostic power in multivariate analysis.

**Conclusions:**

We found that N, LNR and LODDS are all related to 5-year OS and DFS with statistical significance, but only LODDS was found to be an independent prognostic factor for OS in multivariate analysis.

## Background

Adequate study of lymph nodes status is a fundament of right evaluation of the prognostic outcome for patients who undergo surgery for colonic neoplasm so that the common staging system currently used, the 7th edition of TNM by American Joint Committee on Cancer (AJCC), proposes parameter N for nodal evaluation [1]. In particular, N considers the number of lymph nodes involved by metastatic diffusion of tumoural cells on histological examination, without any mention about the total number of retrieved nodes.

Twelve is considered the minimum number of lymph nodes necessary to retain reliable tumour staging, but several factors can potentially influence the lymph node harvesting, such as patient and tumour-related factors, but also pathological analysis and surgical technique. For example, lymph node yield is frequently reduced in male, elderly, obese and lower socioeconomic patients, while large and poorly differentiated tumors, longer resection specimens and pre-operative tumour tattooing are associated with increased lymph node yield [2].

Emergency surgery for complicated colon cancer can represent a trouble for the surgeon, who could come across the necessity to balance the importance of an adequate oncologic resection with the opportunity of a quick control of the septic source and obtaining the hemodynamic stabilization of the patient [3, 4]. This is why emergency surgery can be an obstacle to reach the benchmark of 12 nodes with an accurate lymphadenectomy, in addition to all other variables that can influence the nodal harvesting.

In order to improve the accuracy of the nodal study, a new classification of lymphatic involvement has been proposed, not only in colorectal cancer.

Lymph nodes ratio (LNR) is defined as the ratio between positive lymph nodes and the total number of retrieved lymph nodes. It has been valued for some types of neoplasms such as the stomach, pancreas, bladder and breast, and some studies found that it is more precise than the parameter N in the prognostic evaluation in colorectal cancer [5, 6]. Otherwise, some authors sustain that LNR accuracy is not so reliable when the number of retrieved lymph nodes is low [7]. Furthermore, LNR does not show any advantage over N in case of no lymph node involved in metastatic diffusion, since the ratio is evermore 0.

Another classification system has been recently proposed, that is log odds of positive lymph nodes (LODDS), defined as the logarithm of the ratio between the positive and the negative lymph nodes (more 0.5 both to the numerator and to the denominator, to avoid null values). It has been tested in gastric, breast and colorectal cancer [8], and it seems to have a stronger predictive value on survival than N and TNM [9–11].

To our knowledge, none of the published papers about these new classification systems of nodal involvement in colorectal cancer has been so far performed on a patient who underwent urgent surgery. Our study is the first one carried out on a selected population of patients subjected to urgent surgery for complicated colonic cancer.

## Methods

This is a retrospective study performed on patients who underwent urgent colon resection in Arcispedale Sant’Anna of Ferrara for complicated colonic cancer, confirmed by histological examination, between 1 January 2003 and 31 December 2013. Complications included bowel obstruction, perforation and massive bleeding on any other acute condition such as sepsis due to elapsing conditions (for example, appendicitis, diverticulitis, suppuration of the neoplastic lump). In case of bowel obstruction, we considered only patients with evident signs of occlusion, leaving outpatients treated with medical therapy or endoscopy “bridge to surgery”.

We collected personal data (age, sex), clinical data (ASA score, surgical indication, time and type of surgery, expertise of the surgeon, tumour localization, clinical stage of neoplasm), pathological data (length of surgical specimen, histology, level of infiltration, grading and lymph nodal status) and data about follow-up (possible relapsing, that is loco-regional recurrence or onset of distant metastases, death, overall and disease-free survival).

Exclusion criteria were presence of metastases at diagnosis (stage IV), presence of neoplastic infiltration on surgical margins or macroscopic residual tumour (not R0 surgery), concomitance of other neoplasms, death into the first postoperative month and insufficient data for analysis.

The patients were distributed according to the current staging system (TNM 7th edition), and nodal status was assessed with histological parameter pN. For each patient, LNR and LODDS were calculated. LNR was defined as the number of positive lymph nodes divided by the number of total examined lymph nodes. LODDS was calculated as log (number of positive nodes + 0.5)/(number of the total examined nodes − positive nodes + 0.5). The patients were stratified in three classes for each parameter, according to the cutoff values reported in previously published studies [11]: LNR0 ≤ 0.05, 0.05 < LNR1 ≤ 0.20 and LNR2 > 0.20; − 1.36 ≤ LODDS0, − 1.36 < LODDS1 ≤ 0.53 and LODDS2 > 0.53.

Survival analysis was performed by Kaplan-Meier curves, and the differences between the curves were verified through log-rank test (Mantel-Cox). Cox’s proportional hazard model was used for univariate and multivariate analyses, considering a significant *p* value < 0.5.

## Results

Between 1 January 2003 and 31 December 2013, 320 patients (165 males, 155 females, median age 76 years) underwent urgent colonic resection for complicated colon cancer in our institute. The 70.62% (*n* = 226) achieved at least 12 lymph nodes harvested, and more than one fourth (29.38%, *n* = 94) did not obtain an adequate study of lymph node status. One hundred eighteen patients were excluded for stage IV (57.63%), non-radical resection (5.08%), presence of other neoplasms (5.93%), death into the first postoperative month (10.17%) and insufficient data (21.19%).

Therefore, our population was represented by 202 patients (98 males, 104 females, median age 76 years). Most of them were classified as ASA 3 (58.25%) or ASA 2 (37.63%). These patients underwent surgery for bowel obstruction in 77.72% of cases, bleeding in 11.39%, perforation in 6.44% and other indications in 4.45% (seven cases of neoplasm suppuration, one acute appendicitis, one acute cholecystitis), and they were submitted to right hemicolectomy in 40.1% of case, left hemicolectomy in 9.41%, sigmoidectomy in 21.29%, segmental colonic resection in 16.34%, Hartmann’s colostomy in 8.42% and other surgery in 4.45% (for example, sub-total colectomy). In 26.73% of cases, viscerolysis was associated, in 11.88% prophylactic cholecystectomy, in 4.95% prophylactic appendicectomy, in 3.96% tenual resection and in 1.98% en bloc resection of other abdominal organs. In 81.19% of cases, the intervention was performed by an expert colorectal surgeon.

The most frequent neoplasm localization was the left side of the colon, in particular, sigma 29.21%, sigmoid rectal junction 10.4%, left colon 6.93% and splenic flexure 5.94%, followed by the right colon with 32.18%, transverse colon 9.41% and hepatic flexure 3.96%. 1.98% the tumour was multifocal.

The mean length of the resected colonic tract was 30.38 cm (range 6–95 cm). The median number of harvested lymph nodes was 17 (range 3–79). In 78.71% (*n* = 159) of cases, at least 12 lymph nodes were examined.

The most frequent histologic type was adenocarcinoma NOS (not otherwise specified, 60.4%), followed by carcinoma with mucinous component (19.31%), mucinous carcinoma (13.86%), ring cell carcinoma (0.99%), mixed histology (1.98%) and other types (3.46%). In 4.06% of cases, the tumour was well differentiated (G1), in 73.1% moderately differentiated (G2) and in 22.84% poorly differentiated (G3).

The following were the patients’ distribution for the clinical stage: stage I 1.98% (*n* = 4), stage II 51.48% (*n* = 104) and stage III 46.53% (*n* = 94).

Survival analysis was performed by Kaplan-Meier curves, and differences between curves were evaluated using the log-rank test. We considered significant *p* values < 0.05. We performed univariate and multivariate analyses using Cox’s proportional hazard model. Both overall survival (OS) and disease-free survival (DFS) were considered. Results are resumed in Table 1 (OS) and Table 2 (DFS).

**Table 1:** OS results

Variables	*n*	%	5-year OS (%)	*p* value (log-rank test)	Univariate analysis (HR, 95% CI)	Multivariate analysis (HR, 95% CI)
Sex				0.3399		
M	98	48.52	46.94			
F	104	51.48	50.96		0.8296 (0.5630–1.2224)	
ASA				**< 0.0001**		
1	1	0.51				
2	73	37.63	64.38		0.1482 (0.002899–7.5709)	
3	113	58.25	43.36		0.3089 (0.006054–15.7561)	
4	7	3.61	14.29		1.2455 (0.01672–92.7711)	
5	0	0				
Surgical indication				**0.0332**		
Occlusion	157	77.72	50.32			
Perforation	13	6.44	23.08		2.4624 (0.9303–6.5173)	
Bleeding	23	11.39	56.52		0.8341 (0.4609–1.5095)	
Other	9	4.45	44.44		1.2497 (0.4689–3.3308)	
Type of surgery				0.0905		
Right hemicolectomy	81	40.1	56.79			
Left hemicolectomy	19	9.41	42.11		1.7368 (0.8361–3.6079)	
Sigmoidectomy	43	21.29	51.16		1.3831 (0.8221–2.3270)	
Segmental resection	33	16.34	42.42		1.4637 (0.8443–2.5375)	
Hartmann	17	8.42	35.29		2.2026 (0.9800–4.9504)	
Other	9	4.45	33.33		2.5408 (0.8140–7.9311)	
Surgeon experience						
Expert	164	81.19				
Not expert	38	18.81				
Istotype				0.816		
Adenocarcinoma NOS	122	60.4	48.36			
Carcinoma with mucinous component	39	19.31	43.59		0.9936 (0.6030–1.6373)	
Mucinous carcinoma	28	13.86	53.57		0.7776 (0.4459–1.3563)	
Signet ring cells carcinoma	2	0.99	50		1.011 (0.1303–7.8436)	
Undifferentiated carcinoma	0	0				
Mixed	4	1.98	50		1.0366 (0.2365–4.5427)	
Other	7	3.46	71.43		0.4153 (0.1597–1.0801)	
G				**0.0274**		
1	8	4.06	75			
2	144	73.1	50.69		2.1613 (0.8548–5.4647)	
3	45	22.84	37.78		3.5917 (1.3088–9.8565)	
T				**0.0024**		
1	0	0				
2	7	3.46	57.14			
3	134	66.34	55.22		0.779 (0.2395–2.5339)	
4a	43	21.29	27.91		1.7467 (0.4998–6.1038)	
4b	18	8.91	50		0.9053 (0.2418–3.3899)	
Stage				**0.0143**		
I	4	1.98	50			
II	104	51.48	57.69		0.6444 (0.1411–2.9434)	
III	94	46.53	39.36		1.1417 (0.2480–5.2562)	
Number of examined lymph nodes				0,0645		
≥ 12	159	78.71	39.53			
< 12	43	21.29	51.57		0.6614 (0.4012–1.0903)	
N				**0.0053**		
0	108	53.46	57.41			
1	61	30.2	42.62		1.5446 (0.9951–2.3975)	
2	33	16.34	33.33		2.1957 (1.2084–3.9896)	
LNR				**< 0.0001**		
0	120	59.41	56.67			
1	49	24.26	48.98		1.1791 (0.7515–1.8499)	
2	33	16.34	21.21		2.9812 (1.5726–5.6517)	
LODDS				**< 0.0001**		
0	89	44.06	62.92			
1	80	39.6	45		1.6684 (1.1060–2.5170)	***p*** **= 0.0251**
						1.7593 (1.0759–2.8767)
2	33	16.34	21.21		3.6757 (1.9145–7.0570)	***p*** **< 0.0001**
						4.2842 (2.4192–7.5869)

The entries in bold are those with statistical significance (*p* < 0.05)

**Table 2:** DFS results

Variables	*n*	%	5-year DFS (%)	*p* value (log-rank test)	Univariate analysis (HR, 95% CI)	Multivariate analysis (HR, 95% CI)
Sex				**0.0139**		
M	98	48.52	65.31			
F	104	51.48	81.73		0.5038 (0.2939–0.8639)	
ASA				0.4824		
1	1	0.51				
2	73	37.63	71.23			
3	113	58.25	76.11		0.9383 (0.5326–1.6531)	
4	7	3.61	71.43		2.2056 (0.2657–18.3117)	
5	0	0				
Surgical indication				**0.0065**		
Occlusion	157	77.72	73.89			
Perforation	13	6.44	46.15		2.9382 (0.8069–10.6997)	
Bleeding	23	11.39	91.3		0.3116 (0.1368–0.7099)	
Other	9	4.45	66.67		1.438 (0.3631–5.6956)	
Type of surgery				0.7739		
Right hemicolectomy	81	40.1	77.78			
Left hemicolectomy	19	9.41	63.16		1.5884 (0.6262–4.0293)	
Sigmoidectomy	43	21.29	76.74		1.2044 (0.5777–2.5113)	
Segmental resection	33	16.34	69.7		1.4012 (0.6457–3.0406)	
Hartmann	17	8.42	70.59		1.7488 (0.5734–5.3333)	
Other	9	4.45	66.67		1.8544 (0.4404–7.8083)	
Surgeon experience				0.1181		
Expert	164	81.19	63.16			
Not expert	38	18.81	76.22		0.6192 (0.3081–1.2443)	
Istotype				0.2991		
Adenocarcinoma NOS	122	60.4	74.59			
Carcinoma with mucinous component	39	19.31	69.23		1.2165 (0.5973–2.4776)	
Mucinous carcinoma	28	13.86	78.57		0.7532 (0.3477–1.6315)	
Signet ring cells carcinoma	2	0.99	50		5.2445 (0.06004–458.1240)	
Undifferentiated carcinoma	0	0				
Mixed	4	1.98	50		2.0194 (0.2771–14.7187)	
Other	7	3.46	85.71		0.4487 (0.1164–1.7303)	
G				0.2498		
1	8	4.06	87.5			
2	144	73.1	75.69		2.0746 (0.5407–7.9603)	
3	45	22.84	68.89		3.2203 (0.7561–13.7156)	
T				**0.0003**		
1	0	0				
2	7	3.46	85.71			
3	134	66.34	81.34		1.3148 (0.3095–5.5851)	
4a	43	21.29	51.16		4.2641 (0.9084–20.0169)	
4b	18	8.91	66.67		2.4171 (0.4551–12.8374)	
Stage				**0.0067**		
I	4	1.98	75			
II	104	51.48	82.69		0.7248 (0.1144–4.5923)	
III	94	46.53	63.83		1.7553 (0.2743–11.2331)	
Number of examined lymph nodes				0.3275		
≥ 12	159	78.71	81.70			
< 12	43	21.29	71.70		1.4498 (0.7441–2.8248)	
N				**0.0007**		
0	108	53.46	82.41			
1	61	30.2	68.85		1.9121 (1.0413–3.5111)	*p* = 0.0729
						1.8505 (0.9478–3.6131)
2	33	16.34	54.55		3.4804 (1.5203–7.9677)	***p*** **= 0.0022**
						3.13 (1.5150–6.4668)
LNR				**0.0001**		
0	120	59.41	80.83			
1	49	24.26	71.43		1.5172 (0.8051–2.8592)	
2	33	16.34	51.52		3.7601 (1.5941–8.8694)	
LODDS				**0.0001**		
0	89	44.06	82.02			
1	80	39.6	73.75		1.5093 (0.8499–2.6803)	
2	33	16.34	51.52		4.0509 (1.6798–9.7690)	

The entries in bold are those with statistical significance (*p* < 0.05)

Mean follow-up was 64 months (range 1–154 months).

Regarding OS (Table 1), significant differences from survival curves emerged for ASA score, surgical indication, tumour grading, T parameter, tumour stage, N parameter, LNR and LODDS. Respectively, OS scores were 57.41% for N0, 42.62% for N1, 33.33% for N2, 56.67% for LNR0, 48.98% for LNR1, 21.21% for LNR2, 62.92% for LODDS0, 45% for LODDS1 and 21.21% for LODDS2. In multivariate analysis, only LODDS was found to be an independent prognostic factor.

Concerning DFS (Table 2), we found significant differences between survival curves of sex, surgical indication, T parameter, tumour stage, N parameter, LNR and LODDS, but none of these confirmed its prognostic power in the multivariate analysis since we found statistical significance for N2 curve only.

## Discussion

Lymph node involvement is one of the most important prognostic factors in colon cancer [1]. An adequate lymphadenectomy is crucial for correct treatment and staging of colonic cancer, but several factors can affect lymph node harvesting [2]. Emergent surgery for complicated colon cancer (perforation, occlusion, bleeding) could represent a further difficulty to perform a correct oncologic resection with an adequate lymphadenectomy. In our experience, more than one fourth of patients (29.38%) did not reach the benchmark of 12 examined lymph nodes. So, an efficient classification system of lymphatic involvement is crucial to define the prognosis, the indication to adjuvant therapy and the follow-up.

LNR seems to be more accurate than N because it is less influenced by a total number of harvested lymph nodes [12], but it is useless in N0 patients.

Some studies showed the prognostic superiority of LODDS compared with N and LNR [9, 10] and its usefulness in node-negative patients [11].

Our study is the first one conducted on a selected population of a patient subjected to urgent surgery for complicated colonic cancer.

**Fig. 1:**
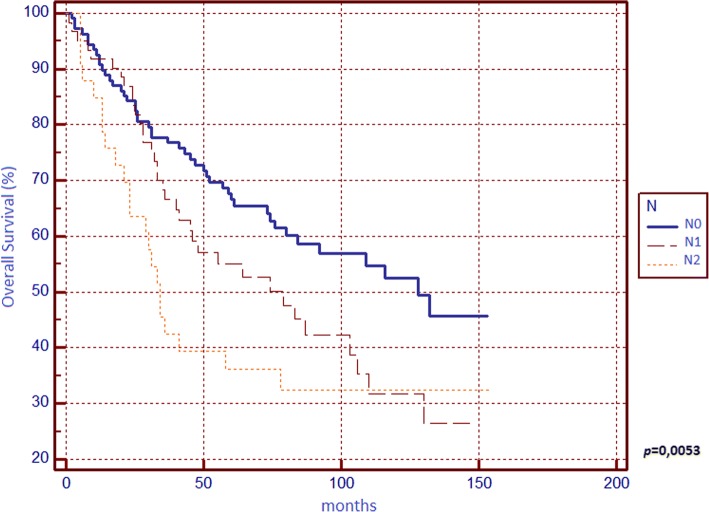
Kaplan-Meier survival curves about overall survival for N0, N1 and N2 patients

**Fig. 2:**
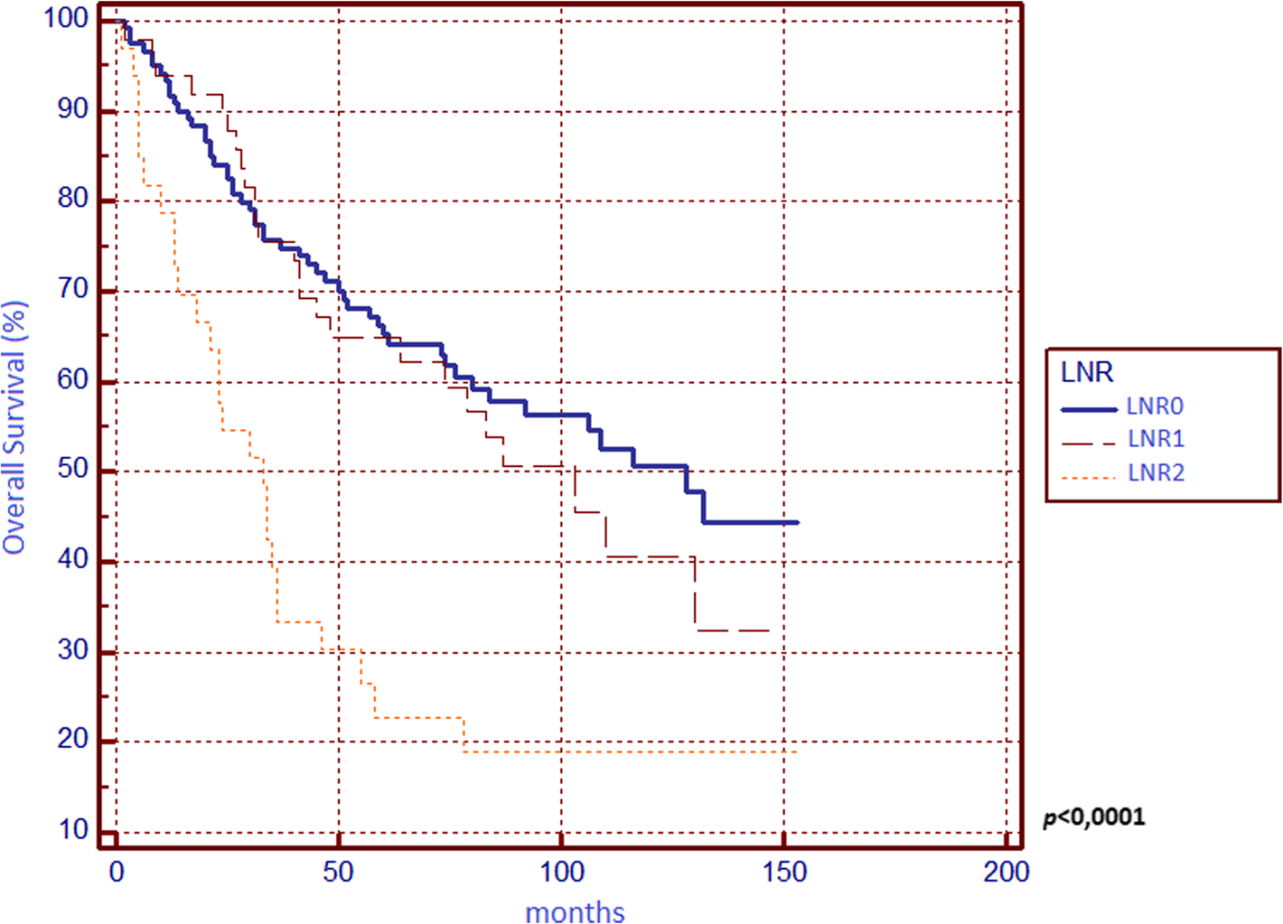
Kaplan-Meier survival curves about overall survival for patients stratified by lymph nodes ratio (LNR)

**Fig. 3:**
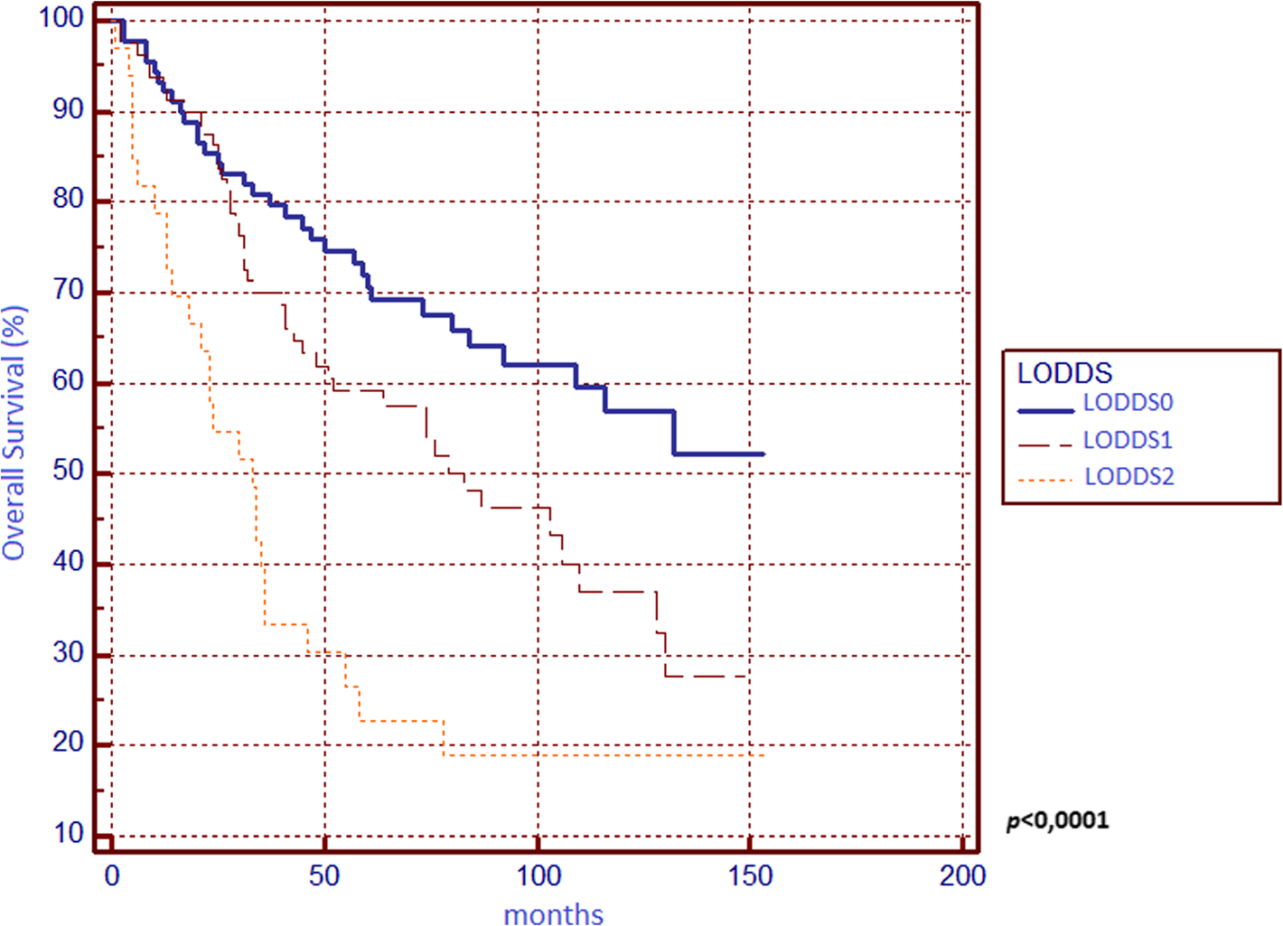
Kaplan-Meier survival curves about overall survival for patients stratified by log odds of positive lymph nodes (LODDS)

**Fig. 4:**
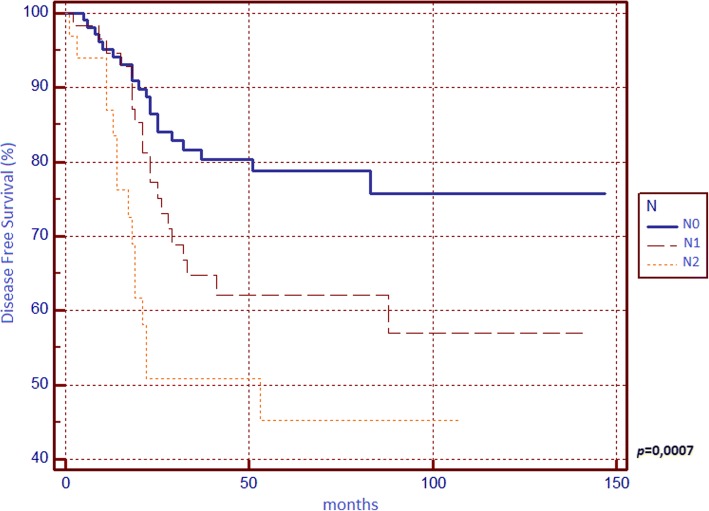
Kaplan-Meier survival curves about disease-free survival for N0, N1 and N2 patients

**Fig. 5:**
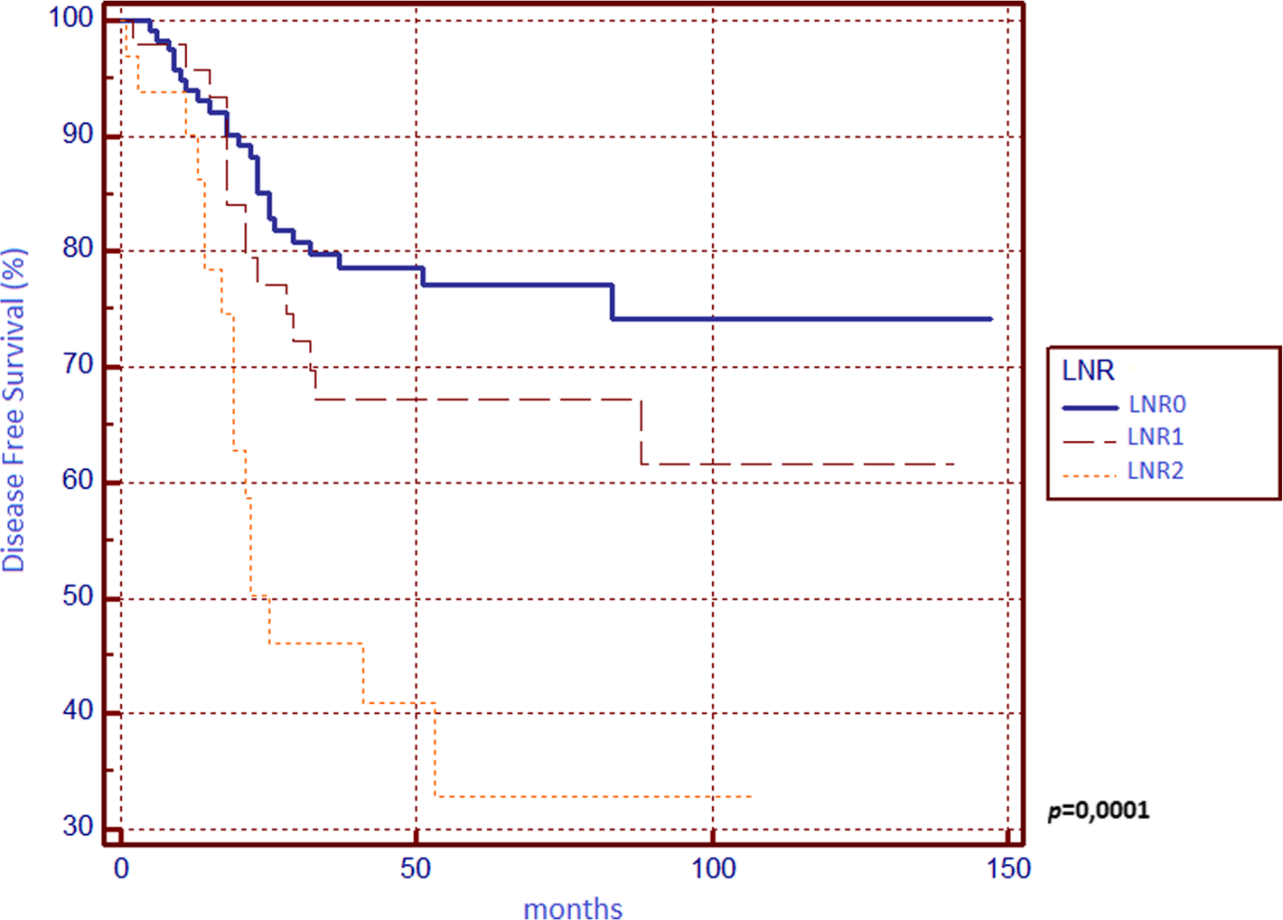
Kaplan-Meier survival curves about disease-free survival for patients stratified by lymph nodes ratio (LNR)

**Fig. 6:**
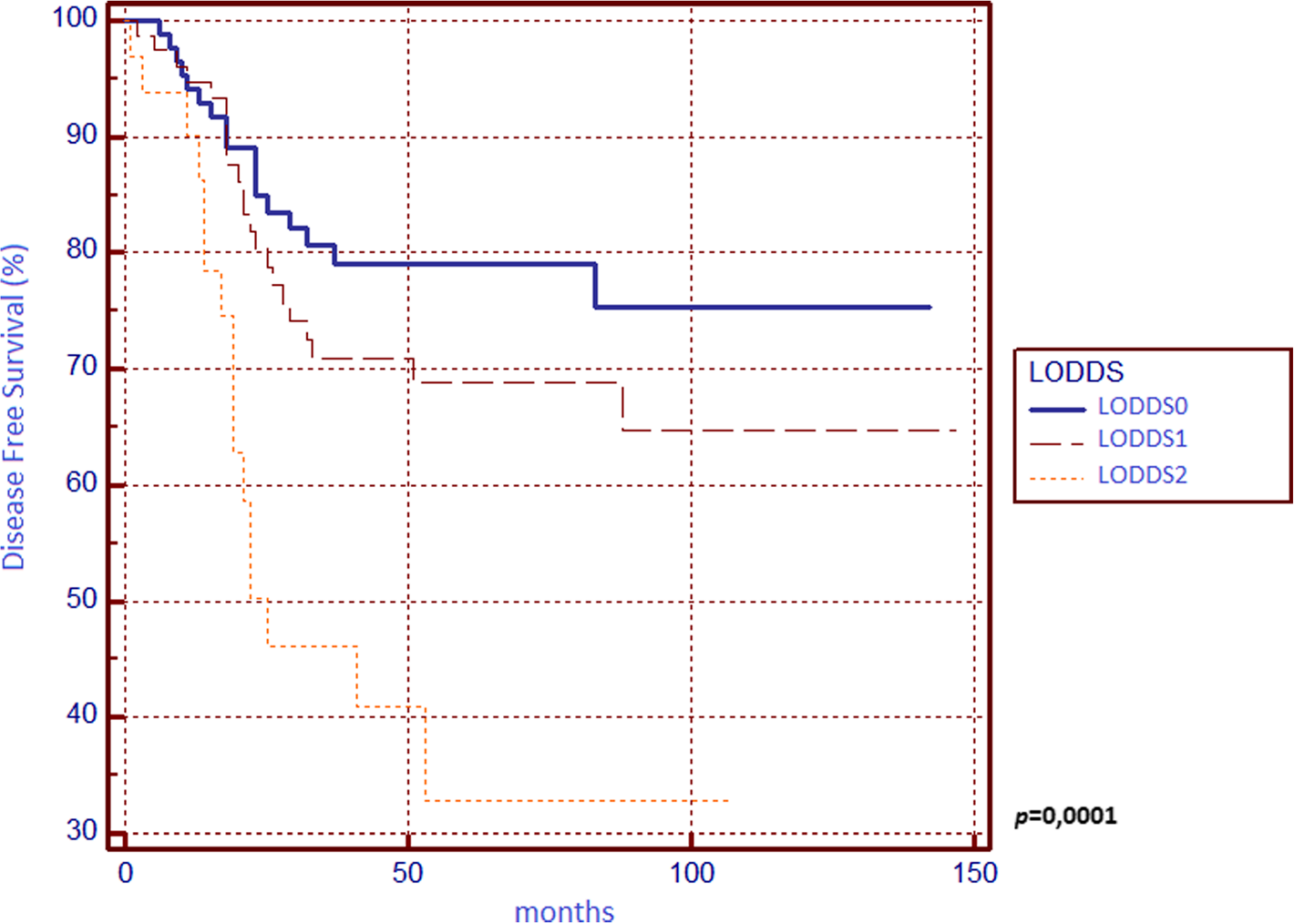
Kaplan-Meier survival curves about disease-free survival for patients stratified by log odds of positive lymph nodes (LODDS)

We found that N, LNR, and LODDS are all related to 5-year OS (Figs. 1, 2 and 3) and DFS (Figs. 4, 5 and 6) with statistical significance, but only LODDS was found to be an independent prognostic factor for OS in multivariate analysis.

In our experience, about one fourth of node-negative patients (24.32%) had less than 12 retrieved lymph nodes. Twenty-six of 111 (23.42%) of N0 and LNR0 patients were classified LODDS1, which converged into a class of higher prognostic risk. Five of 26 (19.23%) patient N0 but LODDS1 experienced a disease recurrence. It is hard to say if these patients could have benefited from an adjuvant therapy, but it is legitimate to think that these patients could have received a more accurate assessment of prognostic risk if LODDS had been considered for the cancer staging.

Our study has some limits. This is a retrospective study carried out on a single-centre database with a limited number of patients, insufficient to draw definitive conclusions.

## Conclusions

Despite the limits of our study, we can conclude that either LNR and LODDS could be included in the prognostic assessment of a patient subjected to urgent surgery for complicated colonic cancer. We found that LODDS is an independent prognostic factor for OS of these patients.

Further studies are necessary to evaluate the efficacy of LNR and LODDS to predict the DFS and to confirm their usefulness in prognostic assessment in urgent colonic surgery.

## Abbreviations

ASA: American Society of Anesthesiologists
DFS: Disease-free survival
LNR: Lymph nodes ratio
LODDS: Log odds of positive lymph nodes
OS: Overall survival
